# Second-order slip effect on bio-convectional viscoelastic nanofluid flow through a stretching cylinder with swimming microorganisms and melting phenomenon

**DOI:** 10.1038/s41598-021-90671-z

**Published:** 2021-05-27

**Authors:** Hassan Waqas, Umar Farooq, Zahir Shah, Poom Kumam, Meshal Shutaywi

**Affiliations:** 1 Department of Mathematics, Government College University Faisalabad, Layyah Campus, Faisalabad, 31200 Pakistan; 2 Department of Mathematical Sciences, University of Lakki Marwat, Lakki Marwat, 28420 Khyber Pakhtunkhwa Pakistan; 3 Center of Excellence in Theoretical and Computational Science (TaCS-CoE), Faculty of Science, King Mongkut’s University of Technology Thonburi (KMUTT), 126 Pracha Uthit Rd., Bang Mod, Thung Khru, Bangkok, 10140 Thailand; 4 Fixed Point Research Laboratory, Fixed Point Theory and Applications Research Group, Center of Excellence in Theoretical and Computational Science (TaCS-CoE), Faculty of Science, King Mongkut’s University of Technology Thonburi (KMUTT), 126 Pracha Uthit Rd., Bang Mod, Thung Khru, Bangkok, 10140 Thailand; 5 Department of Medical Research, China Medical University Hospital, China Medical University, Taichung, 40402 Taiwan; 6 Department of Mathematics College of Science and Arts, King Abdulaziz University, P. O. Box 344, Rabigh, 21911 Saudi Arabia

**Keywords:** Engineering, Mathematics and computing, Physics

## Abstract

The uses of nanofluid in cooling technology is growing. The nanofluid is made up of metallic and nonmetallic particles that are distributed in a base fluid. This research provides a summary of fuel cell models, uses, and how they function. Researchers have made significant contributions in the following era due to the importance of bioconvection in nanotechnology and a variety of biological systems. The idea of the recent work is to evaluate the aspects of the Cattaneo–Christov (C–C) heat and mass flux model, the second-order boundary with melting phenomenon on the bioconvective flow of viscoelastic nanofluid across a cylinder. The nature of the activation energy, thermal conductivity is also taken into account. Appropriate similarity transformations are utilized to reframe the PDEs of the modeled system into a system of ODEs. The governing equations for the renovated system of ODEs are treated by a shooting function. Here bvp4c built-in function computational tool MATLAB is used. The two-dimensional flow has ceased application in several areas, such as polymer industry, material synthesis technology, nano-biopolymer computer graphics processing, industry, mechanical engineering, airplane structures, and scientific research, which is much more useful in nanotechnology. The results of emerging important flow-field parameters are investigated with the aid of graphs and numerical results.

## Introduction

Pertinently, investigators and technologists have carried out many scientific and computational studies to increase the efficiency of industrial applications. Scientists and researchers have experimentally shown that heat transport is necessary for the dominance of multi-scale growth. Continuous phase fluids usually gain thermal properties^1^. Information on the incorporation of materials into continuous phase fluid for the delivery of improved means of transport is then implemented. Nanofluids are a combination of microscopic nanoparticles and liquid bases. Various critical liquids, like water, fuel oil, and ethylene glycol, are used for the manufacture of the flow of nanofluid. It helps to augment the thermal of the fluid and to enhance the rate of heat transformation. Owing to the high potential of nanofluid, it has a range of uses in engineering, including energy processing, wiring, sheet metal, deformation, lubricant, optical fiber processing, heating roll, and cooling. Nanofluids can also be used in a range of vital fields of scientific and technological development, namely nuclear power stations, electronics, bioengineering, and transport. Choi and Eastman^2^ first proposed the concept of nanofluid. Buongiorno^3^ studied the influence of Brownian diffusion and thermophoresis on energy diffusion and mass conversion. Tiwari and Das^4^ also established a simplified model in which the thermophysical properties of volume fraction substances have been investigated. Kuznetsov and Nield^5^ used the Buongiorno model to explain the transfer of thermophoresis motion and Brownian diffusion on the flow of nanofluid that corresponds to the heating vertical surface via the pervious layer, noting that these movements of thermophoresis and Brownian motion result in a reduction of the heat development initiatives via the surface. Shafiq et al.^6^ discussed convective boundary value and thermal slip meaning in 3-D Darcy-Forchheimer nanofluid flowing across the stretching surface. Waini et al.^7^ addressed the issue of steady flow and thermal transition of a porous spinning thin needle in such a nanofluid. Yang et al.^8^ defined the forced convection heat transformation of water/aluminum nanofluid in a rectangular microchannels model. Irfan et al.^9^ reviewed the mathematical method of unsteady Carreau nanoliquids flow via variable conductivity through a bi-directional stretched surface. Albojamal et al.^10^ have proposed numerically nanomaterials aggregation for flow through a partially filled medium due to constant limits on heat flux. Patil et al.^11^ define the continuous nonlinear mixed convection nanofluid flow of a surface layer with hydrogen gas diffusion. Izadi et al.^12^ mathematically investigated the nanofluid heat transfer via an open-cell mechanical heat sink under a uniform heat flux. Many other researchers^13–26^ studied the nanofluid flow with the C–C heat and mass flux. Akinshilo et al.^27^ analyzed the flow and thermal transfer of nanofluids via the converging or diverging channels via the porous tube. The influences of the magnetic field implementation on the heat transmission and entropy output of nanofluids through the triangular microchannels sheet wall were examined by Nguyen et al.^28^. Varzaneh et al.^29^ studied the hydrodynamic and thermal transition parameters of nanoparticles using Numerical simulations in a smooth curved microtube. Bestman^30^ first created an analytical model flowing in the presence of energy linked to chemical reactions on the fitted sheet using a disruption approach. Khan et al.^31^ discussed non-Newtonian material rheology via the use of electromagnetic, MHD nanofluid, and the influence of activation energy. Khan et al.^32^ examined the effect of variable thermal conductivity with Arrhenius activation energy on the 2nd-grade nanofluid flow by nanomaterials.

Bioconvection happens as the normal microbe swims upwards, so the bacteria are denser than the foundation fluids. As the top surface of the base liquid gets so dense due to the multitude of bacteria, it becomes fragile, then the microorganisms decrease and create bioconvection as well as the return of the microbes to swim sustain the bioconvection process. This migration of microorganisms inside the water increases the temperature and the converted mass of the environment as a whole. Microscopic species have played an important role in improving human life, especially because of medical applications. Life is impossible to contribute without the use of microorganisms. Continuum numerical models are built by denying the length of the chambers as well as cell resistance. It is often believed that the distribution of the concentration of nanoparticles is immense relative to the cell axis. Bioconvection happens as biochemical and mixed nanoliquids are treated employing heat and mass conversion. In the first position, Platt^33^ used the term bioconvection and polygonal rotating systems were studied in dense Tetrahymena societies. Kuznetsov^34^ established the important findings for the absorption of nanomaterials. Chu et al.^35^ examined the steady flow of incompressible and two-dimensional laminar results of the non-Newtonian system on the expandable surface by motile microorganisms. Li et al.^36^ investigated the aspects of swimming bioconvection on nanoliquids containing gyrotactic microorganisms and Wu slip characteristics. Nadeem et al.^37^ investigated the efficiency of drag tolerance, heat, and mass transformation in the boundary layer flowing via the density of microorganisms. Khan et al.^38^ studied the relationship of motile microorganisms on the nonlinear mixed convection Magnetohydrodynamic flow of thyrotrophic nanoparticles. Sohail et al.^39^ examined the Maxwell nanoliquids, including the gyrotactic motile microorganism, in the absence of homogeneous means that the model by modified mass and heat flow systems. Elanchezhian et al.^40^ explored gyrotactic microorganism’s effects in bioconvection Oldroyd-B nanofluid past a vertical stretching sheet comprising mixed convection and also a magnetic field inclination. Abbasi et al.^41^ examined the migration of viscoelastic nanoliquids, including a gyrotactic motile microorganism, through a rotating expanding disc with a convective boundary as well as zero mass flow constraints. Also, some relevant studies on the bioconvective model may be linked to research^42–48^.

The main aim of this research work is to evaluate the aspects of the Cattaneo–Christov (C–C) heat and mass flux model, the second-order boundary with melting phenomenon on the bioconvection flow of viscoelastic nanofluid past the cylinder. The nature of the activation energy, thermal conductivity is also taken into account. Appropriate similarity transformations are used to reframe the PDEs of the modeled system into a system of ODEs. The governing equations for the renovated system of ODEs are treated by a shooting function. Here bvp4c built-in function MATLAB computational tool is used. The heat profile decays for a larger estimation of Prandtl number and thermal conductivity parameter. The concentration profile upsurges the magnitude of activation energy and microorganisms profile decreases bioconvection Lewis number. The $Nr$ increasing effect then skin friction is decreased while thermophoresis parameters $Nt$ boosted up effect the Nusselt number increased.

## Mathematical formulation

**Figure 1:**
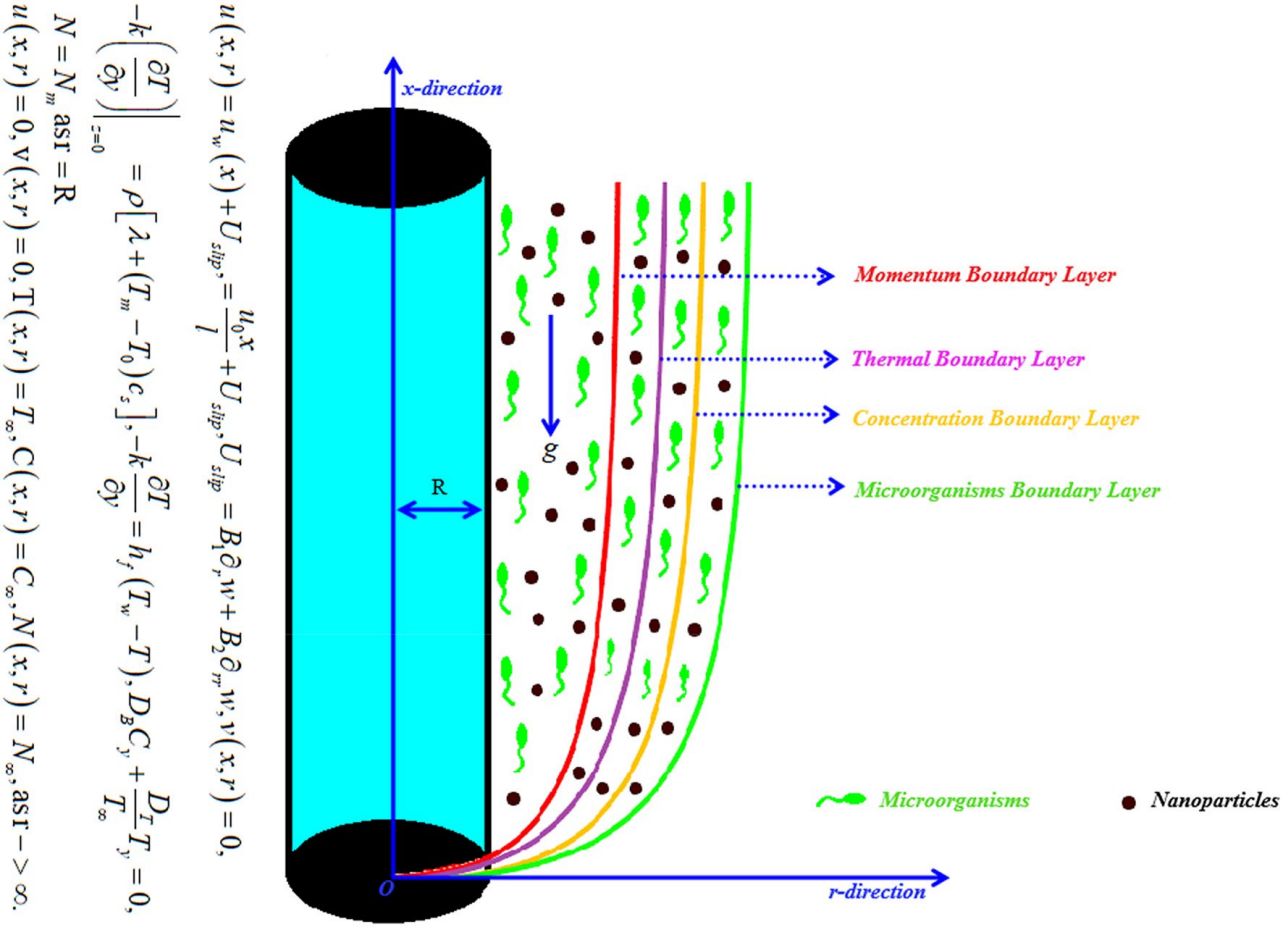
_Geometry of problem_.

This study deals with the 2D Bioconvectional flow of incompressible viscoelastic nanofluid having motile microorganisms via a stretched cylinder with thermal conductivity and activation energy impacts as illustrated in Fig. 1. The Cattaneo–Christov heat and mass flux theory is also considered. The ambient temperature, concentration, and motile microorganisms are symbolized as $T_{\infty } ,\,\,\,C_{\infty }$ and $N_{\infty }$. The physical description of the problem is given below.

The governing equations are^49,50^.

*Continuity equation*

1 $$\frac{{\partial \left( {ru} \right)}}{\partial x} + \frac{{\partial \left( {rv} \right)}}{\partial r} = 0,$$

*Velocity equation*

2 $$\begin{aligned} \frac{{\partial \left( {ru} \right)}}{\partial x} + \frac{{\partial \left( {rv} \right)}}{\partial r} & = v\left( {\frac{{\partial^{2} u}}{{\partial r^{2} }} + \frac{1}{r}\frac{\partial u}{{\partial r}}} \right) + \frac{{\alpha_{1} }}{\rho }\left( {u\frac{{\partial^{3} u}}{{\partial r^{3} }} + u\frac{{\partial^{3} u}}{{\partial x\partial r^{2} }} - \frac{\partial u}{{\partial r}}\frac{{\partial^{2} v}}{{\partial r^{2} }} + \frac{\partial u}{{\partial x}}\frac{{\partial^{2} u}}{{\partial r^{2} }}} \right) \\ & \quad + \frac{{\alpha_{1} }}{\rho }\left( {v\frac{1}{r}\frac{{\partial^{2} u}}{{\partial r^{2} }} + u\frac{{\partial^{2} u}}{\partial x\partial r} - \frac{\partial v}{{\partial r}}\frac{\partial u}{{\partial r}} + \frac{\partial u}{{\partial x}}\frac{\partial u}{{\partial r}}} \right) + \frac{1}{{\rho_{f} }}\left[ \begin{gathered} \left( {1 - C_{\infty } } \right)\rho_{f} \beta^{**} g*\left( {T - T_{\infty } } \right) \hfill \\ - \left( {\rho_{p} - \rho_{f} } \right)g^{*} \left( {C - C_{\infty } } \right) \hfill \\ - \left( {N - N_{\infty } } \right)g^{*} \gamma \left( {\rho_{m} - \rho_{f} } \right) \hfill \\ \end{gathered} \right], \\ \end{aligned}$$

*Temperature equation*

3 $$\begin{aligned} u\frac{\partial T}{{\partial x}} + v\frac{\partial T}{{\partial r}} & = \sigma \left( {\frac{{\partial^{2} T}}{{\partial r^{2} }} + \frac{1}{r}\frac{\partial T}{{\partial r}}} \right) + \frac{1}{{\rho c_{p} }}\frac{\partial }{\partial z}\left[ {k\left( T \right)\frac{\partial T}{{\partial z}}} \right] + \frac{{\rho^{*} C_{p}^{*} }}{{\rho C_{p} }}\left( {D_{B} \frac{\partial C}{{\partial r}}\frac{\partial T}{{\partial r}} + \frac{{D_{T} }}{{T_{\infty } }}\left( {\frac{\partial T}{{\partial r}}} \right)^{2} } \right) \\ & \quad + \Gamma_{E} \phi_{T} , \\ \end{aligned}$$

where,

4 $$\phi_{T} = u^{2} \frac{{\partial^{2} T}}{{\partial x^{2} }} + v^{2} \frac{{\partial^{2} T}}{{\partial r^{2} }} + \left( {u\frac{\partial u}{{\partial x}} + v\frac{\partial u}{{\partial r}}} \right)\frac{\partial T}{{\partial x}} + \left( {u\frac{\partial v}{{\partial x}} + v\frac{\partial v}{{\partial r}}} \right)\frac{\partial T}{{\partial r}} + 2uv\frac{{\partial^{2} T}}{\partial x\partial r},$$

*Concentration equation*

5 $$\begin{aligned} u\frac{\partial C}{{\partial x}} + v\frac{\partial C}{{\partial r}} & = D_{B} \left( {\frac{{\partial^{2} C}}{{\partial r^{2} }} + \frac{1}{r}\frac{\partial C}{{\partial r}}} \right) + \frac{{D_{T} }}{{T_{\infty } }}\left( {\frac{{\partial^{2} T}}{{\partial r^{2} }} + \frac{1}{r}\frac{\partial T}{{\partial r}}} \right) - Kr^{2} \left( {C - C_{\infty } } \right)\left( {\frac{T}{{T_{\infty } }}} \right)^{n} \exp \left( {\frac{{ - E_{a} }}{{K_{1} T}}} \right) \\ & \quad + \Gamma_{C} \phi_{C} , \\ \end{aligned}$$

where

6 $$\phi_{C} = u^{2} \frac{{\partial^{2} C}}{{\partial x^{2} }} + v^{2} \frac{{\partial^{2} C}}{{\partial r^{2} }} + \left( {u\frac{\partial u}{{\partial x}}\frac{\partial C}{{\partial x}} + v\frac{\partial u}{{\partial r}}\frac{\partial C}{{\partial x}}} \right) + 2uv\frac{{\partial^{2} T}}{\partial x\partial r} + \left( {u\frac{\partial v}{{\partial x}}\frac{\partial C}{{\partial r}} + v\frac{\partial v}{{\partial r}}\frac{\partial C}{{\partial r}}} \right),$$

*Microorganism swimming equation*

7 $$u\frac{\partial N}{{\partial x}} + w\frac{\partial N}{{\partial r}} + \left[ {\frac{\partial }{\partial x}\left( {N\frac{\partial C}{{\partial x}}} \right)} \right]\frac{{bW_{c} }}{{\left( {C_{w} - C_{\infty } } \right)}} = D_{m} \frac{\partial }{\partial x}\left( {\frac{\partial N}{{\partial x}}} \right),$$

### Boundary conditions

The following boundary conditions are as follows^48^:

8 $$\left. \begin{gathered} u\left( {x,r} \right) = u_{w} \left( x \right) + U_{slip} , = \frac{{u_{0} x}}{l} + U_{slip} , \hfill \\ U_{slip} = B_{1} \partial_{r} w + B_{2} \partial_{rr} w,v\left( {x,r} \right) = 0, \hfill \\ - k\left. {\left( {\frac{\partial T}{{\partial y}}} \right)} \right|_{\,\,z = 0} = \rho \left[ {\lambda + \left( {T_{m} - T_{0} } \right)c_{s} } \right], \hfill \\ - k\frac{\partial T}{{\partial y}} = h_{f} \left( {T_{w} - T} \right),D_{B} C_{y} + \frac{{D_{T} }}{{T_{\infty } }}T_{y} = 0,N = N_{m} as\,r = R \hfill \\ u\left( {x,r} \right) = 0,v\left( {x,r} \right) = 0,T\left( {x,r} \right) = T_{\infty } , \hfill \\ C\left( {x,r} \right) = C_{\infty } ,N\left( {x,r} \right) = N_{\infty } ,as\,r - > \infty . \hfill \\ \end{gathered} \right\},$$

In the above equations $\left( {u\& v} \right)$ are the velocity components in the direction of $\left( {x\& r} \right)$, $\left( T \right)$ is temperature, $\left( C \right)$ is concentration, $\left( {\beta_{C} } \right)$ the concentration expansion coefficient, $\left( N \right)$ are microorganisms, $\left( g \right)$ gravitational acceleration, $\left( \rho \right)$ signify density of the fluid, $\left( {\beta_{T} } \right)$ the thermal expansion coefficient, $\left( l \right)$ the characteristic length, $\left( {c_{p}^{*} } \right)$ the specific heat of fluid, $\left( n \right)$ the surface temperature, $\left( v \right)$ the kinematic viscosity, $\left( {u_{w} \left( x \right)} \right)$ the stretching velocity, $\left( {\rho^{*} } \right)$ the density of the fluid, $\left( \sigma \right)$ the thermal diffusivity of the fluid, $\left( {c_{p} } \right)$ the specific heat of fluid, $\left( {T_{\infty } } \right)$ the ambient temperature, $\left( \mu \right)$ the dynamic viscosity of the fluid, $\left( {C_{\infty } } \right)$ the ambient concentration, $\left( K \right)$ is the viscoelastic nanofluid parameter, and $\left( {N_{\infty } } \right)$ the ambient microorganisms.

### Similarity transformations

The similarity transformations are as follows^48^:

9 $$\left. \begin{gathered} u = \frac{{u_{0} x}}{l}f^{\prime}\left( \zeta \right),v = - \frac{R}{r}\sqrt {\frac{{u_{0} v}}{l}} f\left( \zeta \right),\zeta = \sqrt {\frac{{u_{0} }}{vl}} \left( {\frac{{r^{2} - R^{2} }}{2R}} \right), \hfill \\ \theta \left( \zeta \right) = \frac{{T - T_{\infty } }}{{T_{w} - T_{\infty } }},\phi \left( \zeta \right) = \frac{{C - C_{\infty } }}{{C_{w} - C_{\infty } }},\chi \left( \zeta \right) = \frac{{N - N_{\infty } }}{{N_{w} - N_{\infty } }}, \hfill \\ \end{gathered} \right\},$$

### Dimensionless governing equations

By after implementing similarity transformation in Eqs. (1–7), we get the nonlinear dimensionless ODEs as:

10 $$\begin{aligned} & \left( {1 + 2\alpha \zeta } \right)f^{\prime\prime\prime} + 2\alpha f^{\prime\prime} + ff^{\prime\prime} - f^{\prime 2} + 4\alpha K\left( {f^{\prime}f^{\prime\prime} - ff^{\prime\prime\prime}} \right) + K\left( {1 + 2\alpha \zeta } \right)\left( {2f^{\prime}f^{\prime\prime\prime} + f^{\prime \prime 2} - ff^{iv} } \right) \\ & \quad + \lambda \left( {\theta - Nr\phi - Nc\chi } \right) = 0, \\ \end{aligned}$$

here $$\alpha \left( { = \frac{1}{R}\sqrt {\frac{{v^{*} l}}{{U_{0} }}} } \right)$$ is the curvature parameter, $$Nr\left( { = \frac{{\left( {\rho_{p} - \rho_{f} } \right)\left( {C_{w} - C_{\infty } } \right)}}{{\left( {1 - C_{\infty } } \right)\left( {T_{w} - T_{\infty } } \right)\beta^{*} }}} \right)$$ is buoyancy ratio parameter, $$Nc\left( { = \frac{{\gamma^{**} \left( {\rho_{m} - \rho_{f} } \right)\left( {N_{w} - N_{\infty } } \right)}}{{\left( {1 - C_{\infty } } \right)\left( {T_{w} - \tilde{T}_{\infty } } \right)\beta }}} \right)$$ is bioconvection Rayleigh number, $$\lambda \left( { = \frac{{\beta^{*} g\left( {1 - C_{\infty } } \right)\left( {T_{w} - T_{\infty } } \right)}}{{\left( {m + 1} \right)u_{e}^{2} }}} \right)$$ is the mixed convection parameter

11 $$\begin{aligned} & \left( {1 + 2 \in \alpha \zeta } \right)\theta^{\prime\prime} + \in \theta^{\prime 2} + 2\alpha \theta^{\prime} + \Pr \left( {f^{\prime}\theta^{\prime} - nf^{\prime}\theta } \right) + \left( {1 + 2\alpha \zeta } \right)\left( {Nb\theta^{\prime}\phi^{\prime} + Nt\theta^{\prime 2} } \right) \\ & \quad - \Pr \delta_{T} \left( {ff^{\prime}\theta^{\prime} + f^{2} \theta^{\prime\prime}} \right) = 0, \\ \end{aligned}$$

here $\Pr \left( { = \frac{v}{\alpha }} \right)$ is the Prandtl number, $Nt\left( { = \frac{{ED_{T} \left( {T_{w} - T_{\infty } } \right)}}{{T_{\infty } \alpha }}} \right)$ is the thermophoresis parameter, $\delta_{T} \left( { = \Gamma_{E} a} \right)$ is thermal relaxation parameter, $Nb\left( { = \frac{{ED_{B} \left( {C_{w} - C_{\infty } } \right)}}{\alpha }} \right)$ is the Brownian motion parameter,

12 $$\begin{aligned} & \left( {1 + 2\alpha \zeta } \right)\phi^{\prime\prime} + 2\alpha \phi^{\prime} + Le\Pr \left( {f\phi^{\prime} - nf^{\prime}\phi } \right) + \frac{Nt}{{Nb}}\left( {\left( {1 + 2\alpha \zeta } \right)\theta^{\prime\prime} + 2\alpha \theta^{\prime}} \right) \\ & \quad - \Pr Le\delta_{C} \left( {ff^{\prime}\phi^{\prime} + f^{2} \phi^{\prime\prime}} \right) - \Pr Le\sigma^{*} \left( {1 + \delta \theta } \right)^{n} \exp \left( {\frac{ - E}{{\left( {1 + \delta \theta } \right)}}} \right)\phi = 0, \\ \end{aligned}$$

here $Le\left( { = \frac{\alpha }{{D_{B} }}} \right)$ is Lewis number, $\delta_{C} \left( { = \Gamma_{C} a} \right)$ is concentration relaxation parameter,

13 $$\left( {1 + 2\alpha \zeta } \right)\chi^{\prime\prime} + 2\alpha \chi^{\prime} + Lb\chi^{\prime}f - Pe\left( {\phi^{\prime\prime}\left( {\chi + \varpi } \right) + \chi^{\prime}\phi^{\prime}} \right) = 0,$$

here $Lb\left( { = \frac{\nu }{{D_{m} }}} \right)$ is bioconvection Lewis number, $Pe\left( { = \frac{{bW_{c} }}{{D_{m} }}} \right)$ is Peclet number.

Through the boundary restrictions

14 $$\left. \begin{gathered} Me\theta \left( 0 \right) + \Pr f\left( 0 \right) = 0,f^{\prime}\left( \zeta \right) = 1 + B_{1} f^{\prime\prime}\left( \zeta \right) + B_{2} f^{\prime\prime\prime}\left( \zeta \right), \hfill \\ \theta^{\prime}\left( 0 \right) = - Bi\left( {1 - \theta \left( 0 \right)} \right),\,\,Nb\phi^{\prime}\left( \zeta \right) + Nt\theta^{\prime}\left( \zeta \right) = 0, \hfill \\ \chi \left( \zeta \right) = 1\,\,at\,\zeta = 0,f^{\prime} \to 0,\theta \to 0,\phi \to 0,\chi \to 0,\,as\,\,\zeta \to \infty , \hfill \\ \end{gathered} \right\},$$

here $Me\left( { = \frac{{c_{p} \left( {T_{\infty } - T_{m} } \right)}}{{\lambda + c_{s} \left( {T_{m} - T_{0} } \right)}}} \right)$ is the melting parameter, $B_{1} \left( { = A\frac{r}{R}\sqrt {\frac{{U_{0} }}{\nu l}} } \right)\,$ which is the first-order velocity slip variable and $B_{2} \left( { = B\,\left( {\frac{{U_{0} }}{\nu l}} \right)\frac{r}{R}} \right)$ be the second-order velocity slip constraints.

### Parameters of the industrial interests

In this division, the physical aspects of the temperature profile, concentration of nanoparticles profile, and gyrotactic microorganisms’ profile. Here $Nu$ is the Nusselt number, $Sh$ is the Sherwood number, and $Sn$ is the local density bioconvective number, respectively.

15 $$\frac{Nu}{{{\text{Re}}_{x}^{{{\raise0.5ex\hbox{$\scriptstyle 1$} \kern-0.1em/\kern-0.15em \lower0.25ex\hbox{$\scriptstyle 2$}}}} }} = - \theta^{\prime}(0),$$

16 $$\frac{Sh}{{{\text{Re}}_{x}^{{{\raise0.5ex\hbox{$\scriptstyle 1$} \kern-0.1em/\kern-0.15em \lower0.25ex\hbox{$\scriptstyle 2$}}}} }} = - \phi^{\prime}\left( 0 \right),$$

17 $$\frac{Sn}{{{\text{Re}}_{x}^{{{\raise0.5ex\hbox{$\scriptstyle 1$} \kern-0.1em/\kern-0.15em \lower0.25ex\hbox{$\scriptstyle 2$}}}} }} = - \chi^{\prime}\left( 0 \right),$$

The local Reynolds number $${\text{Re}}_{x} = {\raise0.7ex\hbox{${u_{e} x}$} \!\mathord{\left/ {\vphantom {{u_{e} x} v}}\right.\kern-\nulldelimiterspace} \!\lower0.7ex\hbox{$v$}}$$.

## Solution methodology

The dimensionless ODEs (10–13) with boundary conditions (14) are resolved mathematically by using the MATLAB computational tools bvp4c mathematical shooting method for various estimations of physical flow parameters. The bvp4c function is a finite difference code that uses the Lobatto-IIIa formula. All the numerical outcomes get in this problem are subjected to an error tolerance $10^{ - 6}$. The system of higher order ODEs is reduced into the first-order ODEs by using the variables given below:

Let

18 $$\left. {\begin{array}{*{20}l} {f = p_{1} ,f^{\prime} = p_{2} ,f^{\prime\prime} = p_{3} ,f^{\prime\prime\prime} = p_{4} ,f^{iv} = p_{4}^{\prime } ,} \hfill \\ {\theta = p_{5} ,\theta^{\prime} = p_{6} ,\theta^{\prime\prime} = p_{6}^{\prime } ,} \hfill \\ {\phi = p_{7} ,\phi^{\prime} = p_{8} ,\phi^{\prime\prime} = p_{8}^{\prime } ,} \hfill \\ {\chi = p_{9} ,\chi^{\prime} = p_{10} ,\chi^{\prime\prime} = p_{10}^{\prime } ,} \hfill \\ \end{array} } \right\},$$

19 $$p_{4}^{\prime } = \frac{\begin{gathered} - \left( {1 + 2\alpha \zeta } \right)p_{4} - 2\alpha p_{3} - p_{1} p_{3} + p_{2}^{2} - 4\alpha K\left( {p_{2} p_{3} - p_{1} p_{4} } \right) \hfill \\ - K\left( {1 + 2\alpha \zeta } \right)\left( {2p_{2} p_{4} + p_{3}^{2} } \right) - \lambda \left( {p_{5} - Nrp_{7} - Ncp_{9} } \right) \hfill \\ \end{gathered} }{{K\left( {1 + 2\alpha \zeta } \right)p_{1} }},$$

20 $$p_{6}^{\prime } = \frac{{ - \in p_{6}^{2} - 2\alpha p_{6} - \Pr \left( {p_{2} p_{6} - np_{2} p_{5} } \right) - \left( {1 + 2\alpha \zeta } \right)\left( {Nbp_{6} p_{8} + Ntp_{8}^{2} } \right) + \Pr \delta_{T} \left( {p_{1} p_{2} p_{6} } \right)}}{{\left( {\left( {1 + 2 \in \alpha \zeta } \right) + \Pr \delta_{T} p_{1}^{2} } \right)}},$$

21 $$p_{8}^{\prime } = \frac{\begin{gathered} - 2\alpha p_{8} - Le\Pr \left( {p_{1} p_{8} - np_{2} p_{7} } \right) - \frac{Nt}{{Nb}}\left( {\left( {1 + 2\alpha \zeta } \right)p_{6}^{\prime } + 2\alpha p_{6} } \right) \hfill \\ + \Pr Le\delta_{C} \left( {p_{1} p_{2} p_{8} } \right) + rLe\sigma^{*} \left( {1 + \delta p_{5} } \right)^{n} \exp \left( {\frac{ - E}{{\left( {1 + \delta p_{5} } \right)}}} \right)p_{7} \hfill \\ \end{gathered} }{{\left( {\left( {1 + 2\alpha \zeta } \right) + \Pr Le\delta_{C} p_{1}^{2} } \right)}},$$

22 $$p_{10}^{\prime } = \frac{{ - 2\alpha p_{10} - Lbp_{10} p_{1} + Pe\left( {p_{8}^{\prime } \left( {p_{9} + \varpi } \right) + p_{10} p_{8} } \right)}}{{\left( {1 + 2\alpha \zeta } \right)}},$$

with,

23 $$\left. \begin{gathered} Mep_{5} \left( 0 \right) + \Pr p_{1} \left( 0 \right) = 0,p_{2} \left( \zeta \right) = 1 + B_{1} p_{3} \left( \zeta \right) + B_{2} p_{4} \left( \zeta \right)\,\,, \hfill \\ p_{6} \left( 0 \right) = - Bi\left( {1 - p_{5} \left( 0 \right)} \right),\,\,Nbp_{8} \left( \zeta \right) + Ntp_{6} \left( \zeta \right) = 0, \hfill \\ p_{9} \left( \zeta \right) = 1\,\,at\,\zeta = 0,p_{2} \to 0,p_{5} \to 0,p_{7} \to 0,p_{9} \to 0,\,as\,\,\zeta \to \infty , \hfill \\ \end{gathered} \right\},$$

## Tabular values and discussion

Table 1 depicted that local skin friction boomed up for various variations *Nr*, *K* and *Nc* while declined *Me* and $\lambda$. Tables 2 and 3 showed that local Nusselt number and local Sherwood number rise for distinguished variations of $\Pr$ and reduces for the estimation of $\alpha$. Table 4 reveals the microorganism density number improved with greater *Lb* and *Pe*. Table 5 showed that when our flow parameters are equal to zero ($Nr = 0$, $\lambda = 0$, $Bi = 0$, $Nc = 0$, $E = 0$, $Lb = 0$, and $Pe = 0$) then it reveals good agreement between current outcomes and previous outcomes.

**Table 1:** Solutions of physical parameters via $- f^{\prime\prime}\left( 0 \right)$.

$\lambda$	$K$	$Nr$	$Nc$	$\alpha$	$B_{1}$	$Me$	$- f^{\prime\prime}\left( 0 \right)$
0.1	0.2	0.5	0.5	0.3	1.0	0.1	0.3272
0.6							0.3266
1.2							0.3257
0.2	0.1						0.3122
	0.3						0.3254
	0.7						0.3367
		0.1					0.3266
		1.0					0.3279
		2.0					0.3294
			0.1				0.3265
			1.0				0.3280
			2.0				0.3293
				0.1			0.3176
				0.4			0.3308
				0.7			0.3370
					2.0		0.3265
					3.0		0.3340
					4.0		0.3399
						0.2	0.3266
						0.5	0.3241
						0.8	0.3194

**Table 2:** Solutions of physical parameters via $- \theta^{\prime}\left( 0 \right)$.

$E$	$Le$	$Me$	$\Pr$	$Nr$	$Nc$	$\alpha$	$Nb$	$Nt$	$\lambda$	$- \theta^{\prime}\left( 0 \right)$
0.2	2.0	0.5	2.0	0.5	0.5	0.3	0.2	0.3	0.2	0.3959
0.4										0.3499
0.6										0.3100
0.1	1.0									0.3998
	1.8									0.3995
	3.0									0.3977
		0.1								0.3744
		0.3								0.3110
		0.7								0.2948
			3.0							0.4946
			4.0							0.5701
			5.0							0.6343
				0.1						0.3993
				1.0						0.3974
				2.0						0.3951
				0.5	0.1					0.3997
					1.0					0.3969
					2.0					0.3938
						0.1				0.4487
						0.4				0.3736
						0.7				0.3038
							0.1			0.3974
							0.4			0.3989
							0.7			0.3991
								0.1		0.4086
								0.4		0.3931
								0.7		0.3761
									0.1	0.3981
									0.6	0.3997
									1.2	0.4016

**Table 3:** Solutions of physical parameters via $\phi^{\prime}\left( 0 \right)$.

$\lambda$	$Le$	$\Pr$	$Nr$	$Nc$	$\alpha$	$Nb$	$Nt$	$Me$	$\phi^{\prime}\left( 0 \right)$
0.1	2.0	2.0	0.5	0.5	0.3	0.2	0.3	0.5	0.5972
0.6									0.5996
1.2									0.6024
0.2	1.0								0.5997
	1.8								0.5981
	3.0								0.5966
		3.0							0.7419
		4.0							0.8551
		5.0							0.9514
			0.1						0.5987
			1.0						0.5960
			2.0						0.5926
				0.1					0.5995
				1.0					0.5954
				2.0					0.5906
					0.1				0.6731
					0.4				0.5601
					0.7				0.4558
						0.1			1.1923
						0.4			0.2992
						0.7			0.1711
							0.1		0.2043
							0.4		0.7862
							0.7		1.3162
								0.1	0.5616
								0.3	0.4665
								0.7	0.4423

**Table 4:** Solutions of physical parameters via $- \chi^{\prime}\left( 0 \right)$.

$Me$	$\lambda$	$Lb$	$Pe$	$Nr$	$Nc$	$\alpha$	$- \chi^{\prime}\left( 0 \right)$
0.2	0.2	2.0	0.1	0.5	0.5	0.3	0.5405
0.5							0.3645
0.7							0.2140
0.1	0.1						0.6014
	0.6						0.6044
	1.2						0.6081
		1.2					0.4515
		1.8					0.5680
		2.6					0.6937
			0.2				0.6496
			0.8				0.9477
			1.6				1.3734
				0.1			0.6034
				1.0			0.6002
				2.0			0.5965
					0.1		0.6041
					1.0		0.5993
					2.0		0.5937
						0.1	0.6392
						0.4	0.5821
						0.7	0.5227

**Table 5:** Validation of current result with the previous result when ($Nr = 0$, $\lambda = 0$, $Bi = 0$, $Nc = 0$, $E = 0$, $Lb = 0$, and $Pe = 0$).

$\Pr$	Hayat et al.^48^	Current results
3.0	0.41113	0.41112
4.0	0.61589	0.61588
5.0	0.79434	0.79435

## Results and discussion

**Figure 2:**
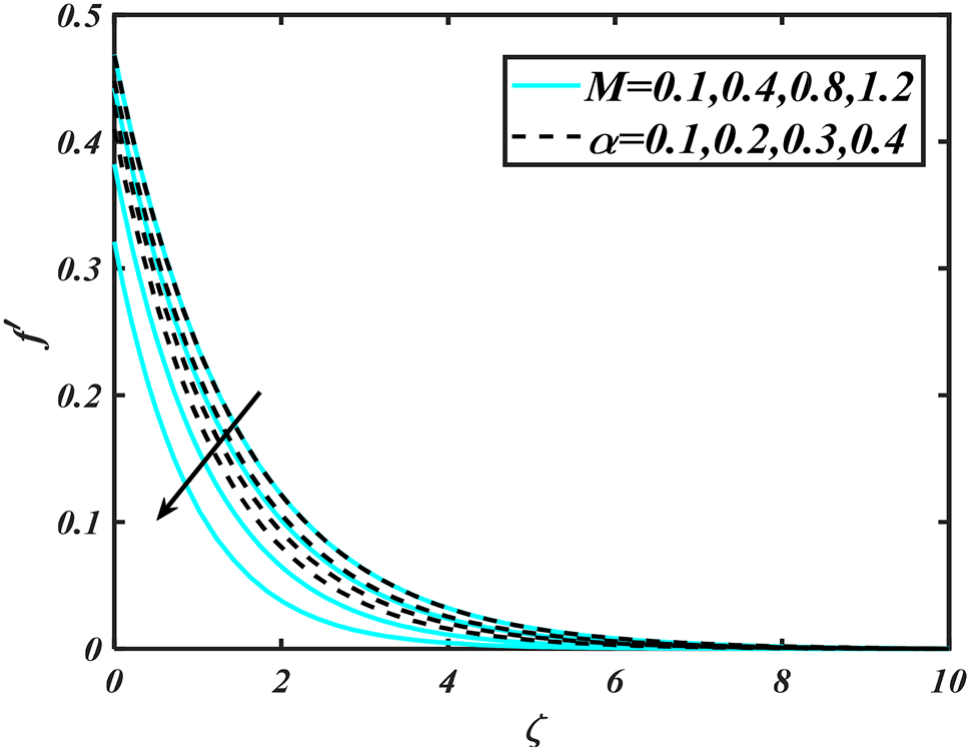
Aspects of $M\,\& \alpha$ versus $f^{\prime}$.

**Figure 3:**
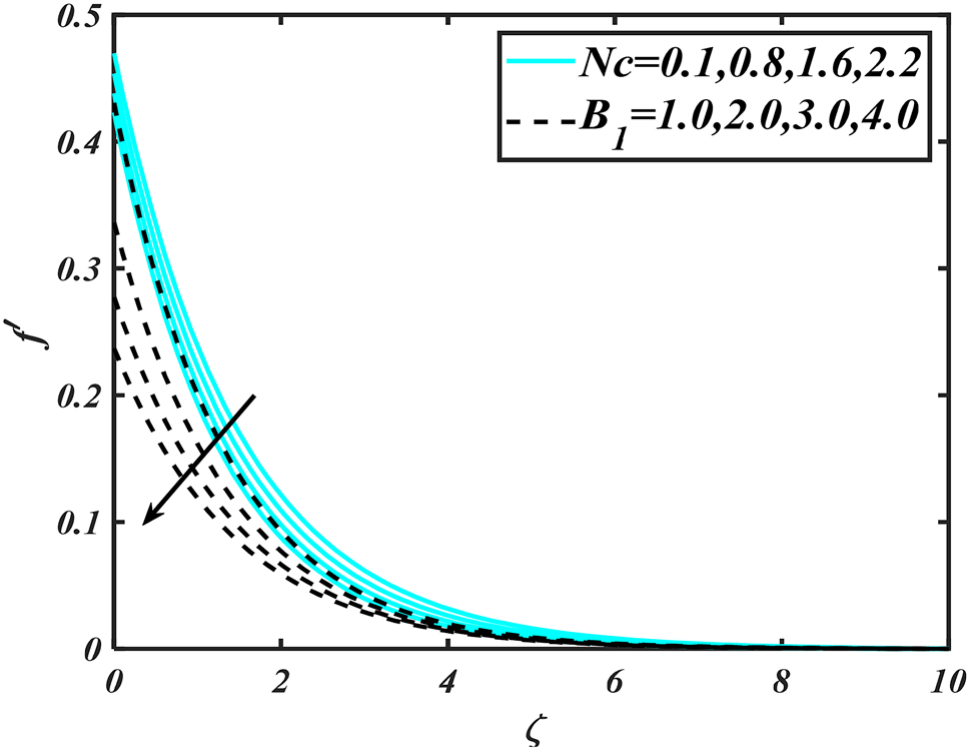
Aspects of $Nc\,\& B_{1}$ versus $f^{\prime}$.

**Figure 4:**
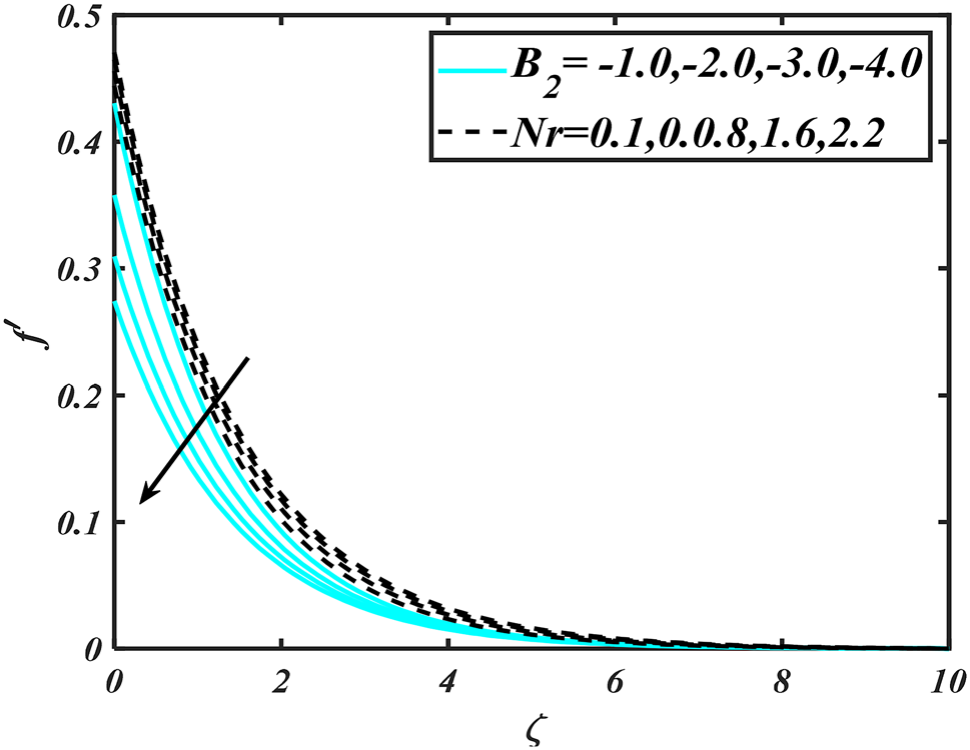
Aspects of $B_{2} \& Nr$ versus $f^{\prime}$.

**Figure 5:**
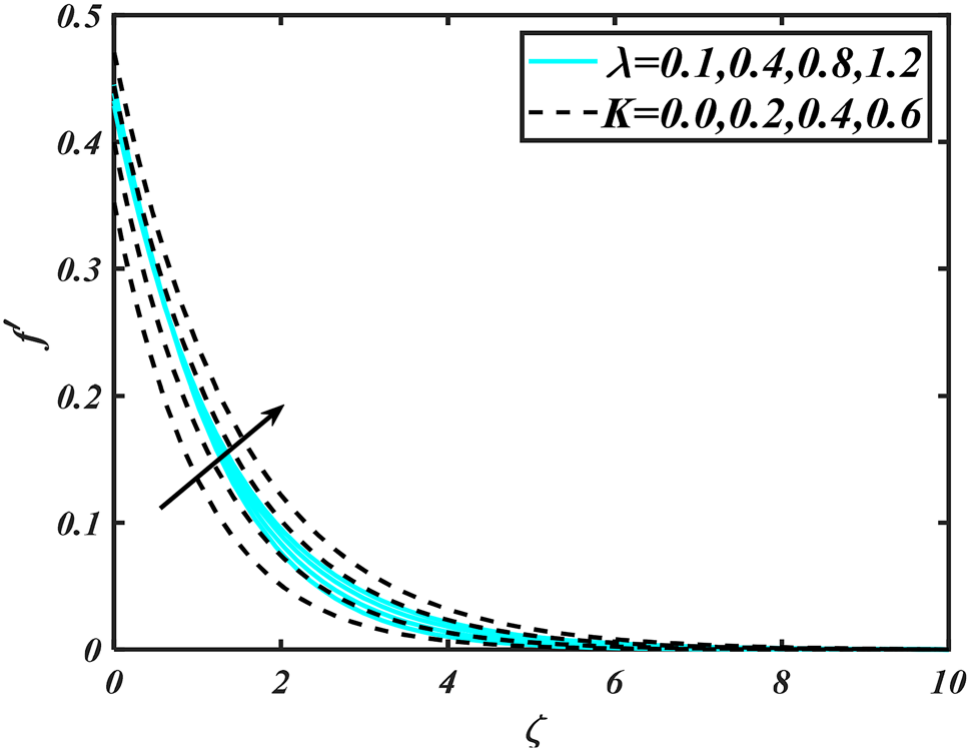
Aspects of $\lambda \,\& K$ versus $f^{\prime}$.

**Figure 6:**
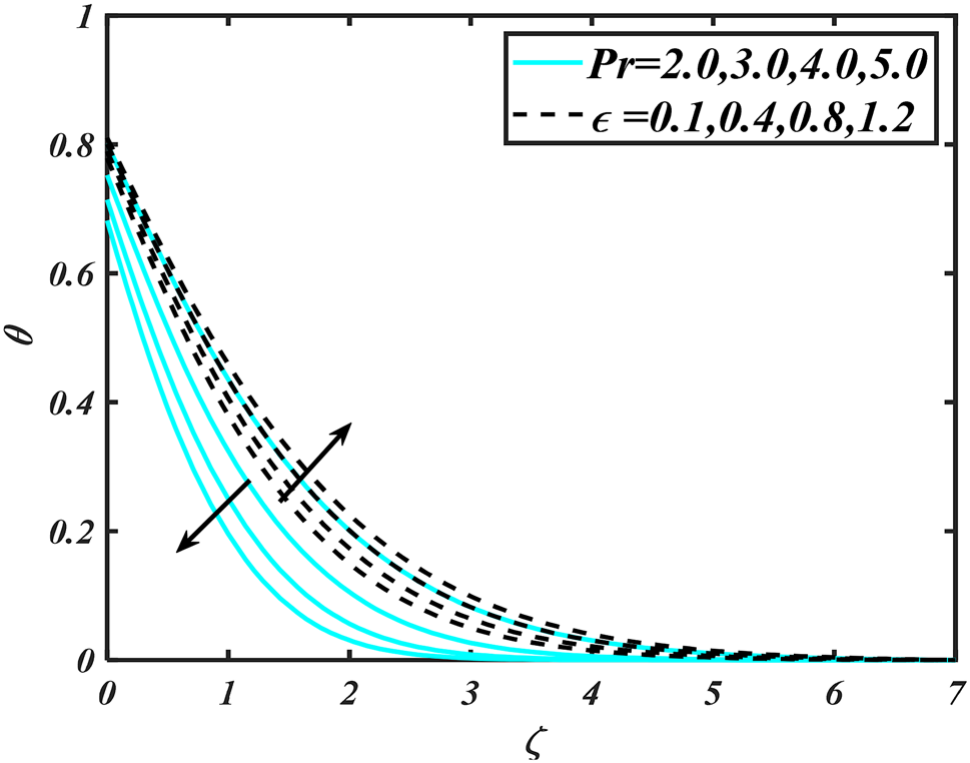
Aspects of $\Pr \,\& \in$ versus $\theta$.

**Figure 7:**
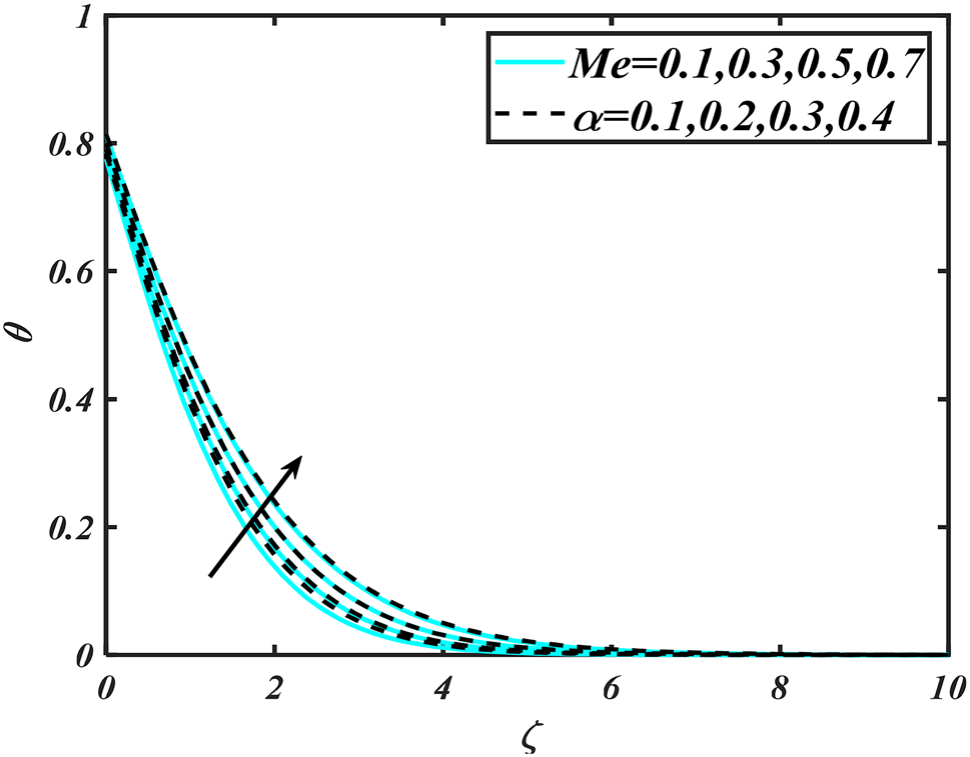
Aspects of $Me\,\& \alpha$ versus $\theta$.

**Figure 8:**
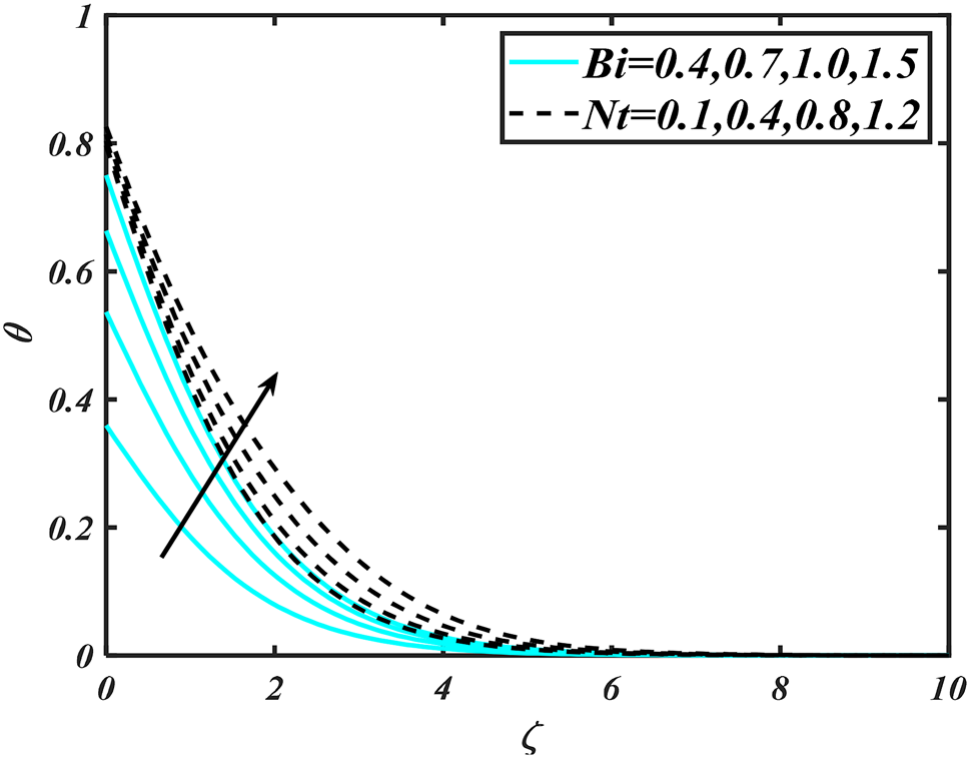
Aspects of $Bi\,\& Nt$ versus $\theta$.

**Figure 9:**
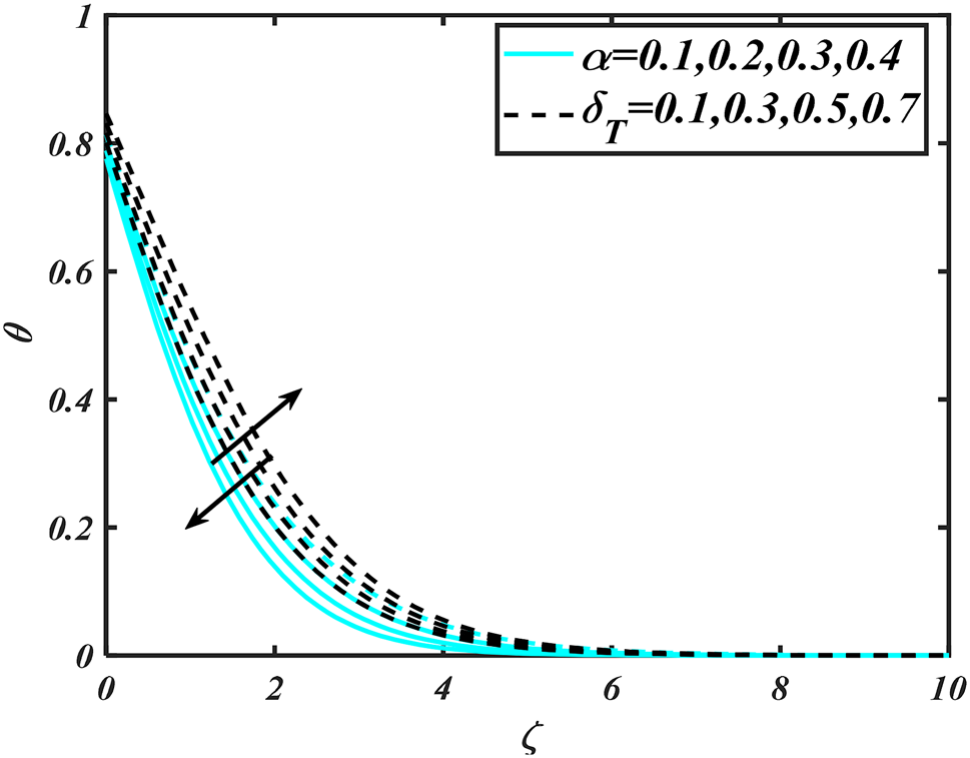
Aspects of $\alpha \,\& \,\delta_{T}$ versus $\theta$.

**Figure 10:**
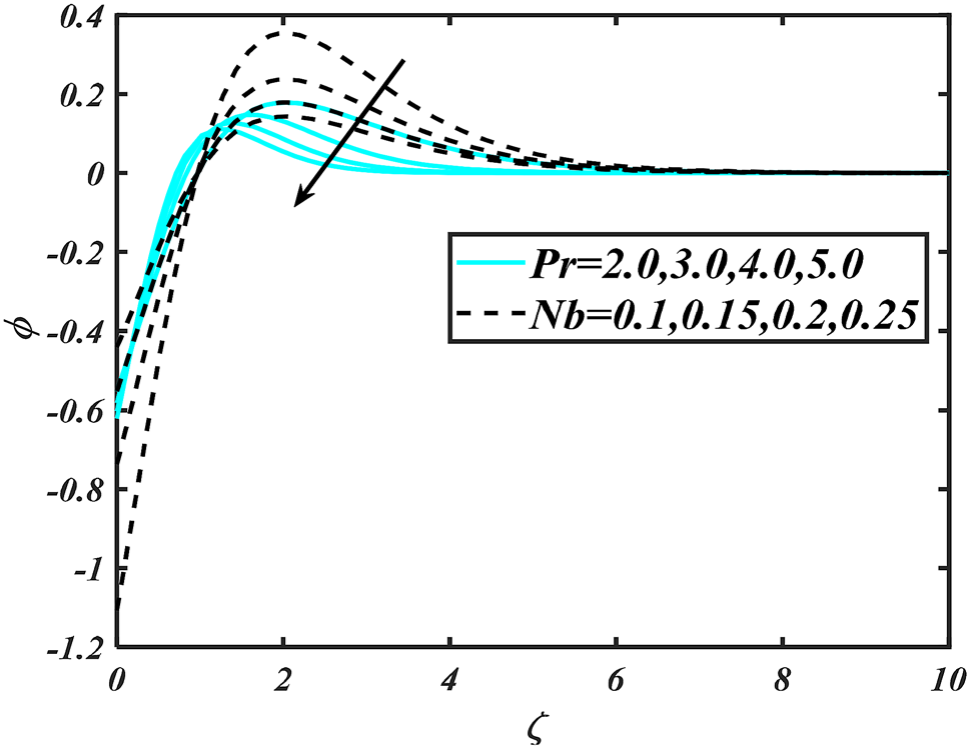
Aspects of $\Pr \,\& Nb$ versus $\phi$.

**Figure 11:**
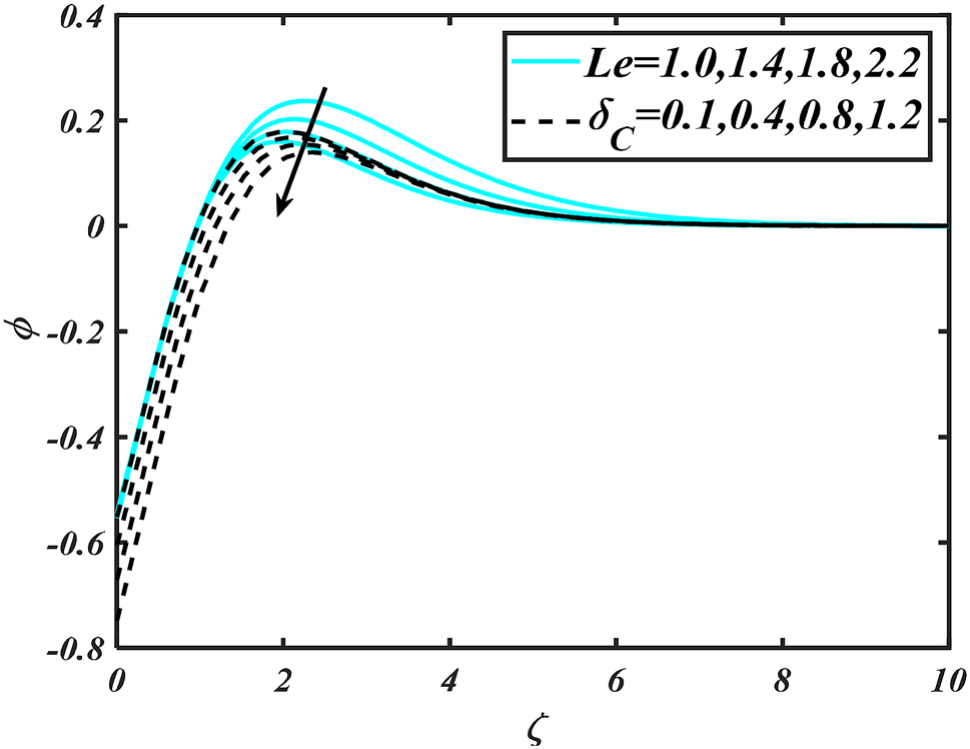
Aspects of $Le\,\& \,\delta_{C}$ versus $\phi$.

**Figure 12:**
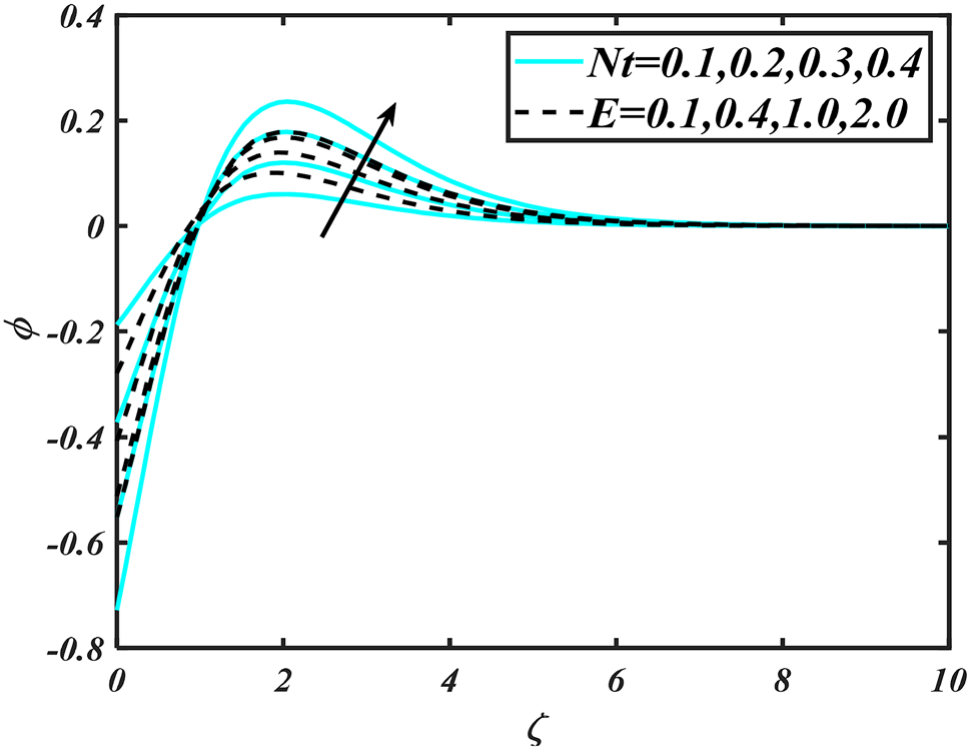
Aspects of $Nt\,\& E$ versus $\phi$.

**Figure 13:**
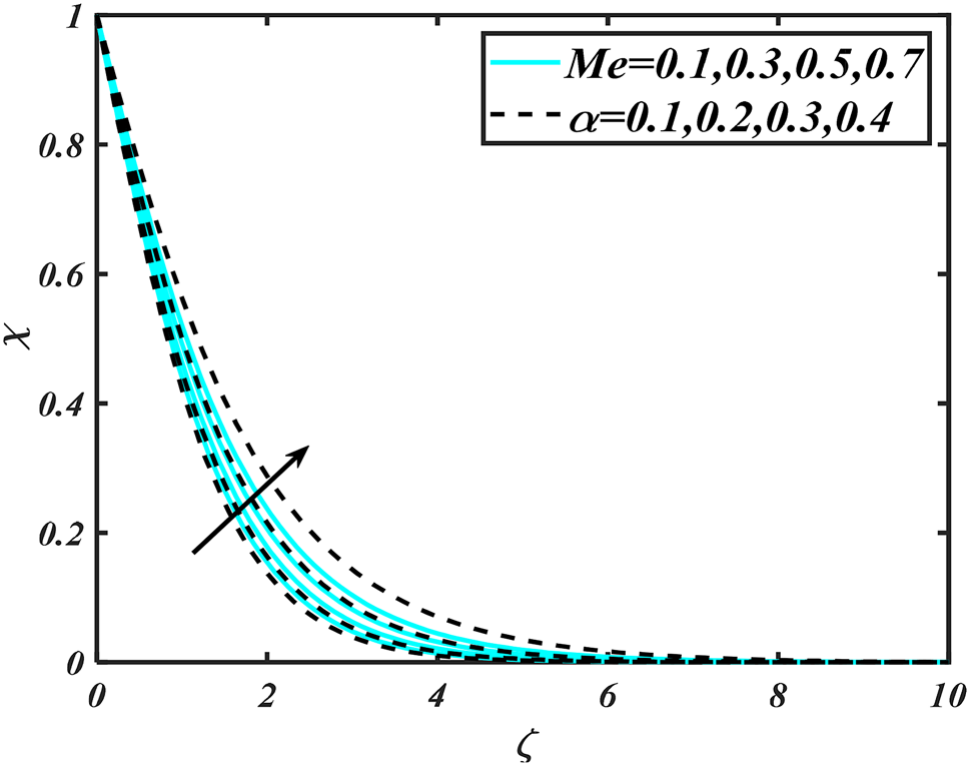
Aspects of $Me\,\& \alpha$ versus $\chi$.

**Figure 14:**
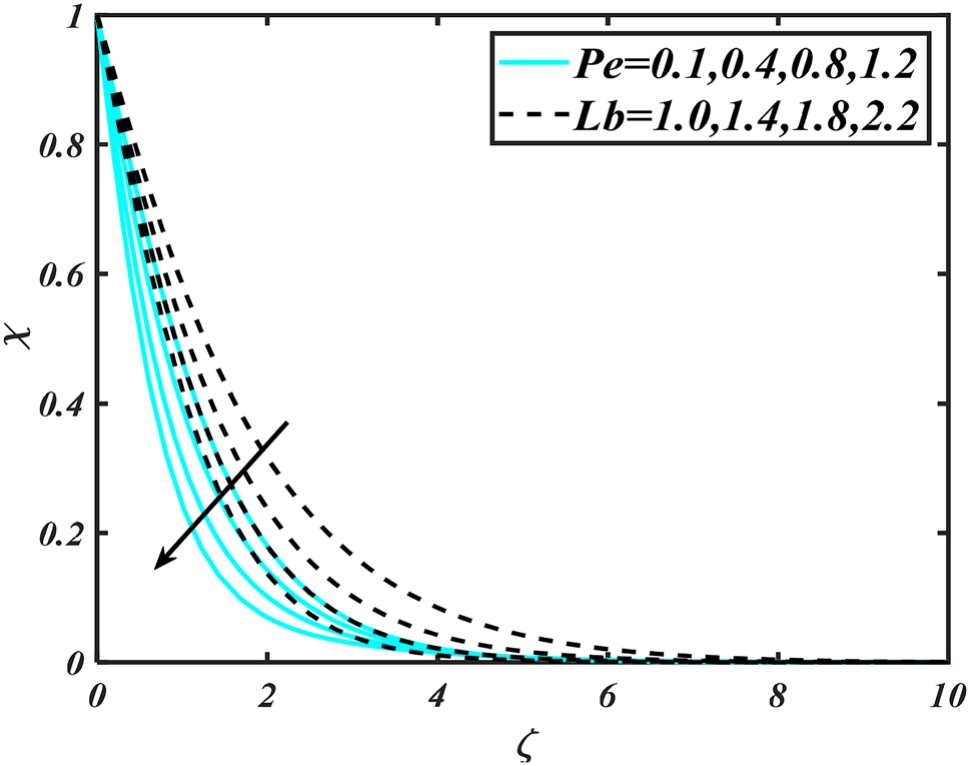
Aspects of $Pe\,\& Lb\,\,$ versus $\chi$.

**Figure 15:**
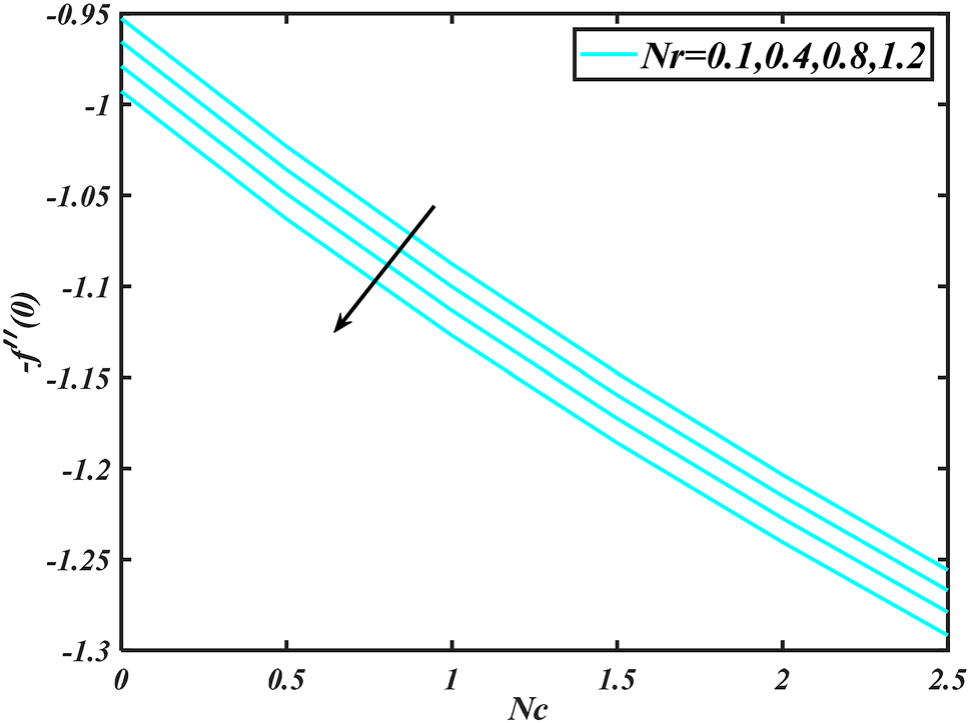
Aspects of $Nr\,\& Nc\,\,$ versus $- f^{\prime}\left( 0 \right)$.

**Figure 16:**
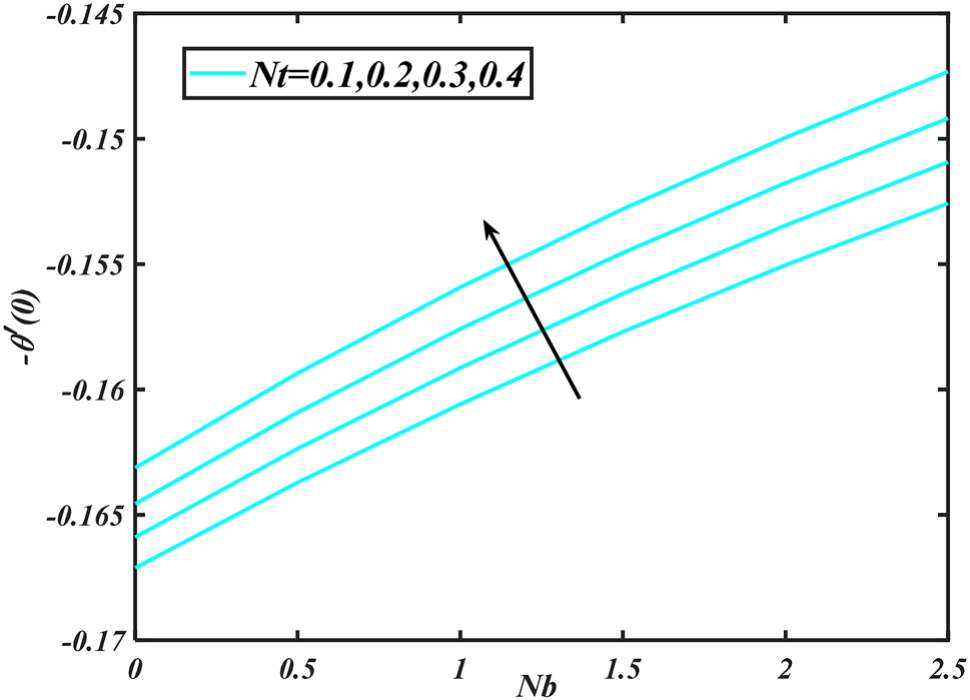
Aspects of $Nt\& Nb$ versus $- \theta^{\prime}\left( 0 \right)$.

**Figure 17:**
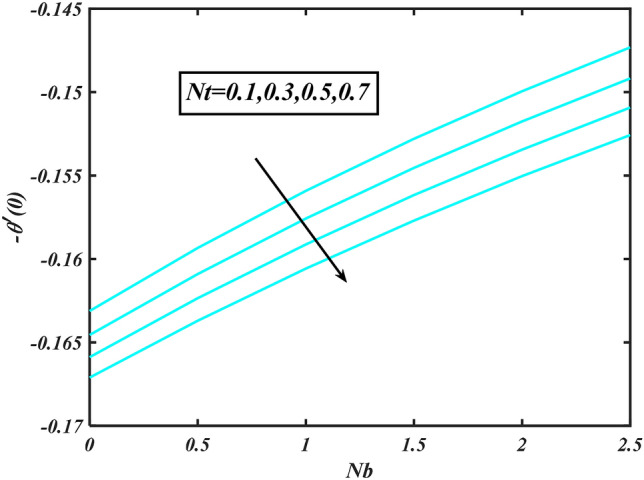
Aspects of $Nt\& Nb$ versus $- \theta^{\prime}\left( 0 \right)$.

**Figure 18:**
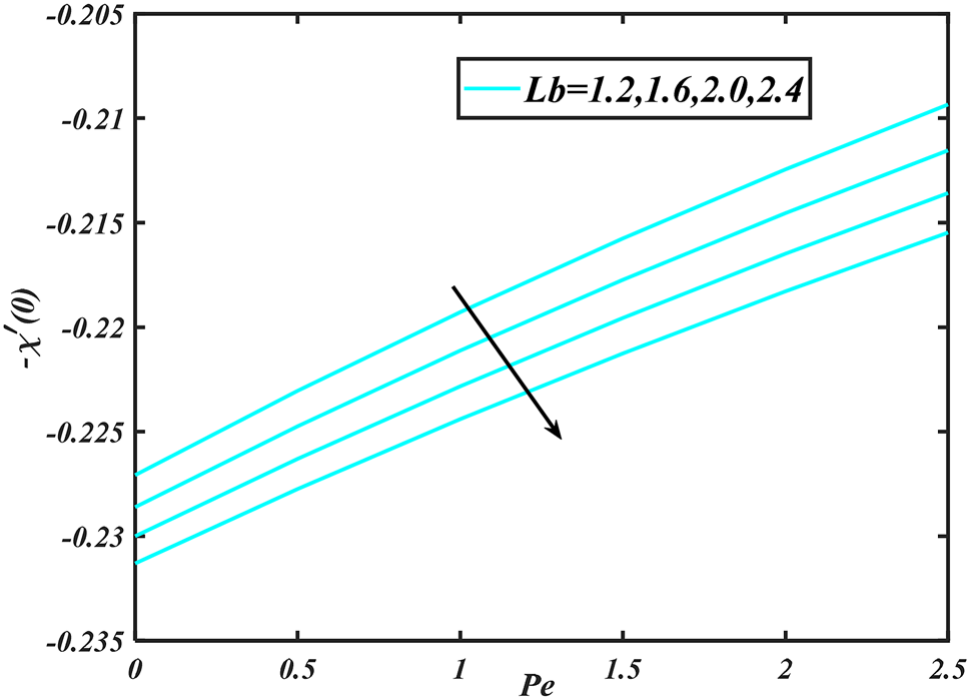
Aspects of $Pe\,\& Lb\,\,$ versus $- \chi^{\prime}\left( 0 \right)$.

In this slice, the significance of this analysis is to explore the properties of 2D Bioconvectional flow of nanofluid through a cylinder in the occurrence of second-order boundary condition and activation energy with melting phenomenon. The aim of this section focuses on the attained numerical result associated with the velocity profile, thermal distribution profile, nanoparticles concentration profile, and motile microorganisms profile for the important involved parameters (thermophoresis parameter, curvature parameter, bioconvection Rayleigh number, mixed convection parameter, Brownian motion parameter, first-order velocity slip, Prandtl number, thermal relaxation parameter, buoyancy ratio parameter, Lewis number, concentration relaxation parameter, bioconvection Lewis number, Peclet number, melting parameter, and second-order velocity slip) that are displayed in Figs. 2, 3, 4, 5, 6, 7, 8, 9, 10, 11, 12, 13, 14, 15, 16, 17 and 18. Similarly to the conventional method, certain fixed values have been assigned to the involved parameters, including such $M = 0.1$, $\alpha = 0.2$, $Nr = 0.2$, $\lambda = 0.5$, $K = 0.2$, $\Pr = 0.7$, $Bi = 2.0$, $Nt = 0.3$, $Nb = 0.2$, $Le = 2.0$, $E = 0.2$, $Lb = 2.0$, and $Pe = 0.1$.

Figure 2 illustrates the effect of magnetic parameter $M$ and curvature parameter $\alpha$ over velocity field $f^{\prime}$. It can be observed that the augmentation of a magnetic parameter $M$ causes diminish velocity of fluid $f^{\prime}$, while observed that the enhancement value of $\alpha$ curvature parameter cause reduction in velocity distribution $f^{\prime}$. Physically this is owing to the magnetic field if a retarding body force recognized as the Lorentz force, which acts transversely in the way of the industrial magnetic profile. The flow of the boundary layer and the thickness of the boundary layer of momentum are declined by the body force. In addition, owing to the resistive force, a fractional resistor force that opposes liquid flow motion, it produced heat. Figure 3 delineated the characteristics of first-order velocity slip $B_{1}$ and bioconvection Rayleigh number $Nc$ against flow of fluid $f^{\prime}$. Since inspected, it is noticed that with higher value of first-order velocity slip $B_{1}$ and bioconvection Rayleigh number $Nc$ the flow of fluid $f^{\prime}$ is reduces. Figure 4 delineated the characteristics of buoyancy ratio parameter $Nr$ and second-order velocity slip $B_{2}$ against the velocity of fluid $f^{\prime}$. Since inspected, it is noticed that with higher value of $Nr$ and second-order velocity slip $B_{2}$ the flow of fluid $f^{\prime}$ is reduces. The difference in both physical parameters is due to buoyancy forces, which aid in raising the temperature of the nanomaterials. Furthermore, the thickness of the thermal boundary is relatively constant. Figure 5 portrays the variation of mixed convection parameter $\lambda$ as well as fluid parameter $K$ via $f^{\prime}$. As expected, it is clear that the velocity distribution $f^{\prime}$ boost due to enlarged mixed convection parameter $\lambda$. Also, it is clear that increasing value of fluid parameter $K$ causes augmentation in the velocity field $f^{\prime}$. Figure 6 depicts the impression of $\Pr$ and thermal conductivity parameter $\in$ on thermal distribution $\theta$. It is witnessed that temperature distribution $\theta$ diminishes with escalating the values of Prandtl number $\Pr$. Physically, higher $\Pr$ values produce less thermal diffusivity, reducing the thermal of the nanofluid. On the other hand, through the greatest value of thermal conductivity parameter $\in$ escalates the temperature distribution $\theta$. Figure 7 shows the consequence of melting parameter $Me$ and curvature parameter $\alpha$ versus temperature distribution $\theta$. It is analyzed that with enlarged value of melting parameter $Me$ as well as curvature parameter $\alpha$ causes upsurges the thermal field of species $\theta$. Figure 8 elucidates the influence of Biot number $Bi$ and thermophoresis parameters $Nt$ over $\theta$ thermal profile. Generally, it is detected that rising magnitudes of Biot number $Bi$ enhanced the thermal field of species $\theta$. And also, with the increment of thermophoresis parameters $Nt$ enhanced the thermal field of species $\theta$. Figure 9 depicts the impression of thermal relaxation parameter $\delta_{T}$ and curvature parameter $\alpha$ on thermal distribution $\theta$. It is witnessed that temperature distribution $\theta$ diminishes with escalating the values of thermal relaxation parameter $\delta_{T}$. On the other hand, through the greatest value of curvature parameter $\alpha$ escalates the temperature distribution $\theta$. Figure 10 is drawn to examine the inspiration of $Nb$ and Prandtl number $\Pr$ versus solutal field of species $\phi$. It is noted that lower solutal field of species is developed by using larger Brownian motion parameter $Nb$ and Prandtl number $\Pr$. The analogous aspects of $\delta_{C}$ concentration relaxation parameter and Lewis number $Le$ via solutal field of species $\phi$ are portrayed in Fig. 11. Here concentration of nanoparticles $\phi$ diminishing by increasing the value of $\delta_{C}$ concentration relaxation parameter and Lewis number $Le$. The influence of thermophoresis parameters $Nt$ and $E$ on volumetric concentration of nanomaterials $\phi$ is depicted in Fig. 12. Remarkably, solutal field $\phi$ of nanoparticles is an enhancing function of thermophoresis parameters $Nt$ and activation energy parameter $E$. The role of activation energy in different chemical processes is significant because it increases the speed of chemical reaction. Additionally, the use of buoyancy effect will help to increase the concentration. Figure 13 is apprehended to show the outcomes of melting parameter $Me$ and curvature parameter $\alpha$ on $\chi$. It is revealing that larger magnitude of melting parameter $Me$ and curvature parameter $\alpha$ microorganism’s field $\chi$ upsurges. The effect of Peclet number $Pe$ and $Lb$ via microorganism field $\chi$ is revealed in Fig. 14. From this scenario it is found that microorganism’s field $\chi$ decline for greater magnitude of $Pe$ and bioconvection Lewis number $Lb$. Figure 15 confirms that growing $Nr$ has an increasing impact on the flow; skin friction is significantly decreased. Figure 16 confirms that rising thermophoresis parameters $Nt$ has an accelerating impact on the flow; Nusselt number is significantly increased. An upsurge in thermophoresis parameters $Nt$, decays Nusselt number, as seen in Fig. 17, whereas a growth in bioconvection Lewis number $Lb$, $- \chi^{\prime}\left( 0 \right)$ is significantly declined as plotted in Fig. 18.

## Final remarks

The study scrutinizes the aspects of thermal conductivity, Fourier and Fick’s laws, and activation energy on the bioconvection viscoelastic nanofluid via a stretching sheet. The second-order boundaries with melting phenomenon are used with appropriate similarity transformations. Mathematical findings computed via shooting scheme with bvp4c (Lobatto-IIIa formula) built-in function MATLAB.

The main points of communication are given below.

- An augmentation in viscoelastic fluid parameters and mixed convection parameter lead to the diminished flow of fluid velocity profile.
- The temperature profile declines for a greater variation of thermal conductivity parameter and Prandtl number.
- The temperature profile improves for larger Biot number and melting parameter while opposite Aspects of curvature parameter.
- The concentration profile of nanoparticles increases with a greater value of activation energy while the opposite trend is analyzed for the Prandtl number.
- The microorganisms profile reduces significantly with booming the variation of bioconvection Lewis number and Peclet number.
